# The Accuracy of the Detection of Body Postures and Movements Using a Physical Activity Monitor in People after a Stroke

**DOI:** 10.3390/s18072167

**Published:** 2018-07-05

**Authors:** Malou H. J. Fanchamps, Herwin L. D. Horemans, Gerard M. Ribbers, Henk J. Stam, Johannes B. J. Bussmann

**Affiliations:** 1 Department of Rehabilitation Medicine, Erasmus MC University Medical Centre Rotterdam, PO Box 2040, 3000 CA Rotterdam, The Netherlands; m.fanchamps@erasmusmc.nl (M.H.J.F.); h.l.d.horemans@erasmusmc.nl (H.L.D.H.); gribbers@rijndam.nl (G.M.R.); h.j.stam@erasmusmc.nl (H.J.S.); 2 Rijndam Rehabilitation, Westersingel 300, 3015 LJ Rotterdam, The Netherlands

**Keywords:** accelerometry, activity monitoring, body postures and movements, physical behavior, stroke, validation

## Abstract

Background: In stroke rehabilitation not only are the levels of physical activity important, but body postures and movements performed during one’s daily-life are also important. This information is provided by a new one-sensor accelerometer that is commercially available, low-cost, and user-friendly. The present study examines the accuracy of this activity monitor (Activ8) in detecting several classes of body postures and movements in people after a stroke. Methods: Twenty-five people after a stroke participated in an activity protocol with either basic activities or daily-life activities performed in a laboratory and/or at home. Participants wore an Activ8 on their less-affected thigh. The primary outcome was the difference in registered time for the merged class “upright position” (standing/walking/running) between the Activ8 and the video recording (the reference method). Secondary analyses focused on classes other than “upright position”. Results: The Activ8 underestimated the merged class “upright position” by 3.8% (775 s). The secondary analyses showed an overestimation of “lying/sitting” (4.5% (569 s)) and of “cycling” (6.5% (206 s)). The differences were lowest for basic activities in the laboratory and highest for daily-life activities at home. Conclusions: The Activ8 is sufficiently accurate in detecting different classes of body postures and movements of people after a stroke during basic activities and daily-life activities in a laboratory and/or at home.

## 1. Introduction

Physical activity is an important component of physical behavior, that is, the body postures, movements and physical activities people perform in daily life [1]. Physical activity, mostly assessed as energy expenditure, has shown to be related to cardiovascular disease, cancer, obesity and fitness [2,3,4]. This applies not only to the general population, but also to people with a chronic condition, such as strokes [5,6].

Many studies focus on overall levels of physical activity. However, physical behavior not only involves an amount of physical activity; for example, it can be described in more detail by frequency, intensity, time and type of activity (that is, the FITT classification) [7]. In older people, it is, for many reasons, more relevant to study other aspects of physical behavior, such as postural allocation and type of activity, than the energy expenditure associated with physical activity [8]. The same reasoning applies to people after a stroke; for example, preventing too much time lying and sitting and promoting upright activities (for example, standing, walking) will prevent deconditioning of the locomotion system and benefit recovery. In other words: the difference between “sitting” and “standing” might be small from the perspective of levels of physical activity and health, but is crucial for the recovery of motor functioning. Therefore, measuring objectively and validly the type of body postures and movements (hereafter called postures/movements) as specific components of physical behavior is needed to assess the functional status and recovery, and to be able to guide and evaluate general and personalized rehabilitation interventions.

Although the number of instruments available to monitor physical behavior is increasing, most have not been validated for the use in people after a stroke. Moreover, these instruments generally focus on overall levels of physical activity or energy expenditure rather than on postures/movements [9]. The few instruments that do detect postures/movements, for example, VitaMove [10,11], PAL2 [12], Dynaport MoveMonitor [13,14], and activPAL [15], are expensive and/or difficult to use in clinical practice.

The Activ8 Physical Activity Monitor (Activ8) [16] is a commercially available one-sensor accelerometer which is unique in the sense that it determines six classes of postures/movements, and energy expenditure (based on movement counts). This monitor is attractive to use in clinical practice because it is user-friendly and low-cost; moreover, it is a noninvasive, small, and lightweight one-sensor monitor. Besides measuring physical behavior, it has a feedback function on the sensor unit itself and results can be shared and discussed on an online communication platform for caregivers and consumers. The Activ8 has shown to be valid for detecting classes of postures/movements in healthy people [17]. However, before the Activ8 can be used to measure physical behavior to assess functional status and recovery in people after a stroke, a population-specific validation study has to be performed. First of all, many people after a stroke have mobility problems as well as a variety of deviating movement patterns, which may lead to misdetection of postures/movements. In addition, in stroke research and treatment, the detection of being upright versus lying or sitting is as important as detecting postures/movements in general. Therefore, the present study examines the accuracy of the Activ8 in detecting several classes of postures/movements during both basic activities and daily-life activities in people after a stroke, performed in a laboratory and/or at home.

## 2. Materials and Methods

### 2.1. Participants

Between October 2015 and February 2016, eligible people after a stroke treated at Rijndam Rehabilitation (Rotterdam, The Netherlands) were invited to participate in this validation study via their physiotherapist or treating physician. For screening, the clinical expertise of the individual’s physical therapist or physician was used. From the perspective of generalization of the results, the selection criteria were kept as broad as possible. Inclusion criteria were: (i) aged 18–75 years; (ii) a history of a stroke; and (iii) mobility problems caused by the stroke. Exclusion criteria were (i) mobility problems not caused by the stroke; (ii) insufficient communication skills or cognitive function to provide informed consent and/or understand the instruction; and (iii) severe mobility problems which would prevent safe participation (that is, functional ambulation category score <3 [18]).

We included 25 people after a stroke: 21 males and 4 females; mean age 56 (standard deviation (SD) 12) years. The mean time post-stroke was 14 (SD 13) months. Of all participants, 16 had an infarction (based on medical records) and 10 were affected on the right side of their body (this was the dominant side in 24 of the 25 participants). The median score on the Berg Balance Scale was 50 (interquartile range 11) [19] with a mean walking speed of 0.8 m/s (SD: 0.4 m/s). All participants gave written informed consent and were assured that they could not be identified via publication (that is, all data were fully anonymized). The study was conducted in accordance with the Declaration of Helsinki, and the protocol was approved by the Medical Ethics Committee of Erasmus MC University Medical Center Rotterdam (MEC 2015-211).

### 2.2. Activ8

The Activ8 (Remedy Distribution Ltd., Valkenswaard, The Netherlands) [16] is a small (30 × 32 × 10 mm) and lightweight (20 g) one-sensor device that contains a triaxial accelerometer, a real-time clock, a battery, and a medium for data storage. The Activ8 determines time spent in the following six classes: “lying/sitting”, “standing”, “walking”, “cycling”, “running”, and “non-wear”. For the primary analyses of this study, the classes “standing”, “walking” and “running” were merged into the class “upright position”. In secondary analyses, “standing” and “walking” were separated, but “walking” and “running” remained merged. The detection of the Activ8 classes is based on the angular position of the sensor and the movement intensity, whereas movement intensity is based on the variability around the mean of the raw acceleration signal. The raw acceleration signals were measured and stored at 12.5 Hz and converted to postures/movements with a resolution of 1.6 Hz. Data were stored with the smallest possible epoch of 5 s, resulting in 8 non-time-stamped samples per 5 s. This characteristic allows us to define how much time a specific Activ8 class was determined in a 5-s epoch, but not (in case of two or more Activ8 classes) in which part(s) of the epoch.

**Figure 1:**
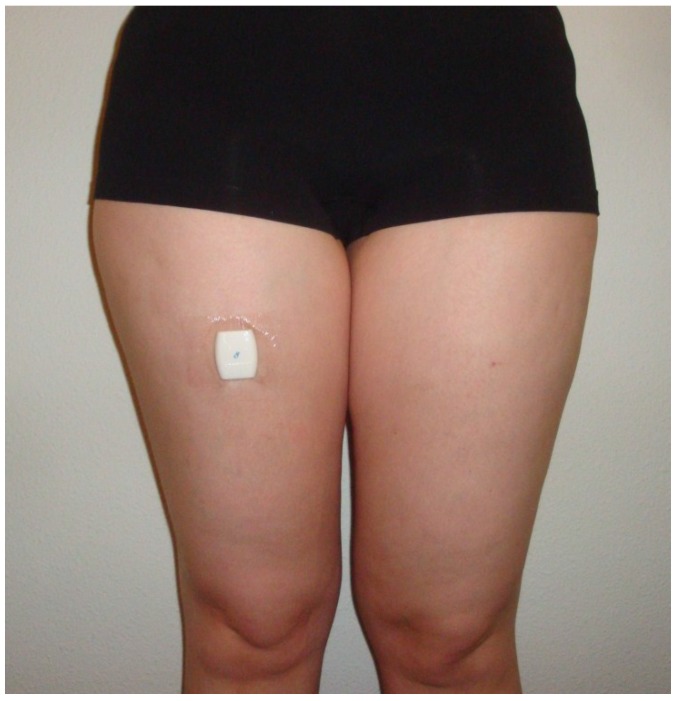
The Activ8 attached to the front of the less-affected thigh.

In the present study, the commercially available professional version was used and attached with Tegaderm™ skin tape to the front of the less-affected thigh, halfway between the hip and knee for the duration of the assessment (Figure 1). This is different from manufacturer’s instructions to place the Activ8 in a trouser pocket. This was done because previous measurements showed that this position can be applied in a more standardized way, would improve the accuracy of detection, and because not everyone has trouser pockets. Participants reported no negative influence from the Tegaderm™ skin tape in terms of the wearing comfort of the Activ8. It is a non-allergic medical skin tape which can be used for several days without complications.

### 2.3. Protocol

Participants were assessed for maximally 1 h; during this period they performed a pre-set protocol with either basic activities or daily-life activities (Table 1). Basic activities included, for example, normal sitting, standing and walking, that is, activities involving one posture/movement only, whereas daily-life activities combined two postures/movements or whole-body movements, for example, vacuuming, unloading the dishwasher, getting dressed, and so forth. The assessment was performed in the laboratory of a rehabilitation center, or at home. Which protocol the participants performed was based on their individual physical ability (that is, more severely affected participants performed basic activities) and on their location (that is, inpatients were assessed at the laboratory). In addition to the pre-set protocol, participants were asked which activities they regularly perform and were not included in the protocol. Those activities were added to the pre-set protocol as the free-choice activities. For the basic activities, participants were instructed to stay as still and as comfortable as possible. The pace for comfortable over-ground walking, walking on a treadmill, and cycling was chosen by the participant, as was the pace of the slower and faster types of walking and cycling. The daily-life activities and free-choice activities were also performed in the participant’s normal way and pace. During all activities, supervision was available to ensure the participant’s safety; however, to enable all activities to be performed as “normally” as possible (to reflect everyday life), supervision was kept as unobtrusive as possible. Based on pragmatic reasons (for example, the physical ability of each individual) some activities were excluded from the protocol. Each activity was scheduled to last approximately 80 s. However, the duration of some activities was either shorter (for example, when the activity was quickly completed), or sometimes longer (for example, when walking or cycling outside) when a participant was assessed at home or performed daily-life activities. A total assessment lasted maximally for 1 h (irrespective of the activities or location) and all participants were given the opportunity to rest between activities. All activities were recorded with a handheld digital video camera which served as the reference method. Video data were synchronized in time with the Activ8 registration.

### 2.4. Data Analyses

To analyze the video recording, clear criteria to score the different video classes were developed, extensively discussed, and applied. To allow more insight into potential error sources, the initial video classes differed from the Activ8 classes. Ten video classes were defined: “lying”, “sitting”, “sit-to-stand transfer”, “standing”, “standing with leg movements”, “shuffling”, “walking”, “staircase walking”, “cycling”, and “running”. Each second of the video recording was assigned to one of these classes. In the case that a class was unclear, a consensus was obtained together with a second researcher. For the main analysis, some video classes were merged. For the primary analysis the following categories were merged: (i) “lying” and “sitting” (lying/sitting class); and (ii) “standing”, “standing with leg movements”, “shuffling”, “walking”, “staircase walking”, “running”, and “sit-to-stand transfer” were merged into the “upright position” class. For the secondary analyses the following categories were merged: (i) “lying” and “sitting” (lying/sitting class); (ii) “sit-to-stand transfer” and “standing” (standing class); and (iii) “shuffling”, “staircase walking”, “walking”, and “running” (walking class). The video class “standing with leg movements” was merged into the “standing” class for the basic standing activities, and into “walking” for the walking activities. For daily-life activities, generally characterized by quick alternations of standing and walking, it was found to be difficult to allocate “standing with leg movements” to the “standing” or “walking” video class. Therefore, for these activities, the secondary analyses with “standing” and “walking” separated were not performed. 

The entire duration of each performed activity was analyzed, except for some samples at the beginning or end when an activity started or ended within a 5-s epoch of the Activ8. Because it was not possible to determine the timing of samples within a 5-s epoch and, as a result, to calculate a 1-s agreement for activities which existed of more than one posture/movement, we compared the total duration of all classes within an activity. The primary outcome was the total difference in time between the Activ8 and the video recording for the class “upright position” (standing/walking/running) presented for all data together (overall data) and for the basic activities and daily-life activities in the laboratory and at home separately. Secondarily, we focused on the total time differences between the Activ8 and the video recording for the two components of “upright position” (the classes standing and walking/running) and for the classes other than “upright position”. The total time difference was defined as the total time of a posture/movement measured by the Activ8 minus the actual time of that posture/movement according to the video recording. This difference was expressed as percentages as well by dividing it by the actual time according to the video recording. We defined a total time difference of 10% as acceptable for both the basic activities and daily-life activities. We used a percentage of 10% because a previous developed activity monitor detecting postures/movements had comparable accuracy and provided meaningful outcomes in previous research [20,21]. Next to the total time difference, the agreement between the Activ8 and the video recording was calculated and defined as the time of a posture/movement correctly classified by the Activ8. To visualize the individual differences in the total time difference, Bland–Altman plots were made. For the classes “upright position”, “lying/sitting”, and “cycling” all the data were used (that is, basic and daily-life activities in the laboratory and at home), whereas, for the classes “standing” and “walking/running”, only data from the basic activities (performed in the laboratory or at home) were used. The reason for this was the described issue of not being able to validly allocate the video class “standing with leg movements” to the “standing” or “walking” video class during daily-life activities. However, to give some insight into the classification of standing and walking during daily-life activities, some exemplary data will be provided per daily-life activity.

## 3. Results

A total of 25 participants were included in this study. Eight of these participants were willing to participate in a second assessment to either perform other activities (basic and daily-life activities) or to perform activities at a different location (laboratory/home). Table 1 presents the number of unique participants included per activity in each of the locations (numbers between brackets). The large differences in the number of participants per activity are due to the ability of the participants; the availability of a bed, staircase, bike, and so forth; and/or due to limited time.

### 3.1. “Upright Position”

**Figure 2:**
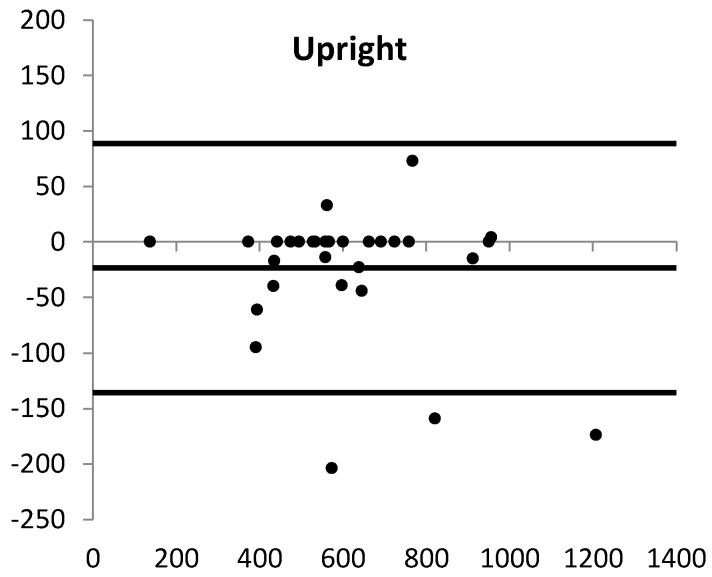

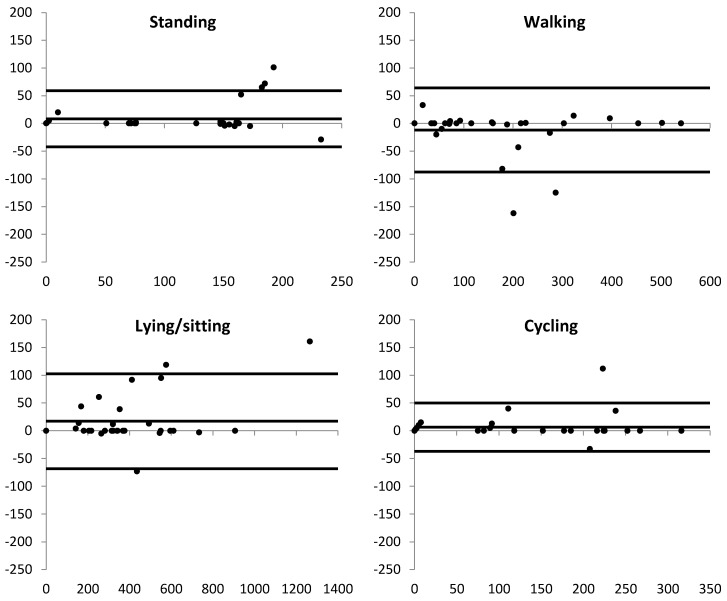
The Bland–Altman plots (x-axis: mean of Activ8 and video recording; y-axis: difference, calculated as Activ8 minus video recording) of the body postures and movements for all data together. The solid lines are the mean difference and the upper and lower limits of agreement. For “standing” and “walking/running”, only data from the basic activities (performed in the laboratory or at home) were used. Each dot is one assessment.

The main results are presented in Table 2. Overall, the Activ8 underestimated the class “upright position” (difference Activ8 versus video −3.8%), ranging from −0.5 to −7.0% for the different activity protocols at the different locations. Figure 2 shows that the total difference between the Activ8 and the video recording was due to differences in about half of the participants of which in most of them “upright position” was underestimated. The agreement between the Activ8 and the video recording ranged from 82.2 to 97.6%.

### 3.2. Components of “Upright Position”

In the basic activities, analysis of the components of “upright position” shows that the Activ8 overestimated the class “standing” (difference Activ8 versus video: laboratory 14.3%; home 1.3%) and underestimated the class “walking/running” (difference Activ8 versus video: laboratory −7.5%; home −6.1%) (Table 2). The variability in the individual differences between the Activ8 and the video recording for “standing” was due to differences in about half of the participants, although dominated by four participants. For “walking/running”, more participants had a difference between the Activ8 and the video recording, but it was here dominated by four participants as well, of which three were the same as those for “standing”. Table 3 (part A) shows the classification of the Activ8 and of the video recording for a part of the daily-life activities in the laboratory. The video class “standing with leg movements” was more or less equal to the difference between the Activ8 classification and the video classification of the class “walking”.

### 3.3. Other Postures/Movements and Free-Choice Activities

Overall, the Activ8 overestimated the duration of “lying/sitting” by 4.5%, resulting from differences in about half of the participants of which in most of them “lying/sitting” was overestimated. For “cycling”, the overestimation was 6.5%, half of this difference was caused by one participant (Table 2; Figure 2). Results of the free-choice activities are presented in Table 3 (part B). At the laboratory, six participants performed the activity in which they propelled a wheelchair with their leg. This was detected by the Activ8 as both “sitting” and “cycling”. One participant had a stair lift at home and the use of this was detected almost entirely as “sitting”. Another participant performed fitness activities on a daily basis at home, for example, stepping up/down on the stairs, and squatting activities; both of these activities were mainly detected as “walking”.

## 4. Discussion

The aim of the present study was to determine the accuracy of the Activ8 to detect several classes of postures/movements in people after a stroke while performing different pre-set activities in a laboratory or at home. A small overall difference in time was found between the Activ8 and the video recording for the main outcome class “upright position”. The difference was smallest for basic activities performed in the laboratory and largest for daily-life activities performed at home. However, this was expected because of the more variation in postures/movements, that is, these are more natural, fluent, and adapted to the activity and environment during daily-life activities and at home. The findings of this study indicate that the Activ8 yields valid measurements of several classes of postures/movements in daily life in people who have suffered a stroke.

Although the “upright position” was generally well detected, it was slightly underestimated by the Activ8: in eight participants, standing (for a shorter or longer period) was classified as “sitting”, and in six, partly the same, participants some walking time was classified as “cycling”. All outliers in the plot of “upright position”, “lying/sitting”, and “cycling” of Figure 2 were part of these participants. This misdetection occurred mainly in participants who stood or walked with flexed hips and, hence, whose thigh was less vertical during these activities. This misdetection is, at least partly, the result of our positioning of the Activ8, that is, we attached the device to the front of the thigh in order to standardize the measurements and avoid non-wear when applied in daily-life measurements. Initially, however, the device was developed to be carried in a trouser pocket, and the signal processing and settings were optimized for this situation. In a previous study, however, the results were more reliable when the device was fixed to the thigh instead of using the trouser pocket [17]. That previous study was performed in healthy people who did not stand or walk with flexed hips. After all these measurements, the manufacturer made it possible to select the sensor location when initializing a measurement, which allows measuring with slightly different settings in the algorithm when the sensor is attached to the thigh, which will lower the above-mentioned errors.

To obtain more insight into the accuracy of the detection of the class “upright position”, its two components “standing” and “walking/running” were examined separately as well. During basic activities, both in the laboratory and at home, the overall standing time was overestimated and the walking time was underestimated. The Activ8 determines the distinction between “standing” and “walking” by a threshold applied to the variability in the acceleration signal, which represents the intensity of the movement. Three participants had a very low walking speed and step frequency (0.14–0.36 m/s; 14–24 steps/min), and the movement intensity of their walking was below the threshold for “walking”. Two of these participants were an outlier of Figure 2. As a result, for those who walk very slowly after the stroke, the Activ8 may not be sufficiently accurate to detect walking. A lower intensity threshold for walking might solve this issue. However, in daily-life activities, the video class “standing with leg movements” seemed to be classified as walking by the Activ8. Although it is debatable whether this is good or not because the leg movement was sometimes very minor, lowering the intensity threshold would also increase classifying such minor leg movements as walking. Overall, it can be concluded that the Activ8 validly detects that someone is in an upright position and that the Activ8 can be used for that during rehabilitation of people after a stroke to assess the functional status and recovery. However, for the distinction between standing and walking, some validity issues remain.

The free-choice activities were included to get an indication of the Activ8 output during “other” activities. One activity was “leg propulsion of a wheelchair”; this was selected because this is a common way for people after a stroke to move around during rehabilitation. In the present study, this activity was classified as “cycling” almost 70% of the time. This misclassification is understandable because the wheelchair driver propels the wheelchair in a sitting position, moving the legs in a way similar to that when cycling. From an energy expenditure perspective, it is debatable whether this misclassification is a problem. However, it does mean that it is difficult to distinguish between actual cycling (for example, on a home trainer during therapy) and propelling a wheelchair with the legs, which could be important when measuring physical behavior during rehabilitation after a stroke. Therefore, it might be helpful to use an additional “short and simple” activity log book.

Overall, the results provided by the Activ8 in people after a stroke were similar to those acquired with the Activ8 in healthy subjects [17] and with the few other activity monitors measuring postures/movements that have been validated for people after a stroke, such as PAL 2 [12] and activPAL [22]. The latter device has also been tested in slow walking people after a stroke, and also showed a decrease in accuracy for walking speeds lower than 0.4 m/s (0.39 to 0.2 m/s: ±70% accuracy; <0.19 m/s: ±55% accuracy) [23]. However, comparing devices on the basis of the literature requires caution due to differences between the studies in terms of activity protocols and study design. Although we realize that other instruments can be used to measure physical behavior in people after a stroke, some important points need to be considered. First, most devices provide overall physical activity data (movement counts, energy expenditure), but no information about postures/movements. Secondly, very few instruments have been validated in people after a stroke. Due to possible changed movement patterns, valid results in healthy people or other patient groups cannot be automatically extrapolated to the stroke population. Together with the ability to give real-time feedback, we believe that the Activ8 has the potential to have additional scientific, practical, and clinical value.

### Study Limitations

The present study has some limitations. First, the total number of samples analyzed was not large due to both the protocol (that is, a selection of representative daily-life activities of short duration) and the participants (that is, not all were able to perform all the pre-set activities). Secondly, it was, unfortunately, not possible to calculate a 1-s agreement for activities which existed of more than one posture/movement. The smallest possible interval is a 5-s epoch, in which it was not possible to determine the timing of the samples. Although the broad inclusion criteria might be a limitation as well, a broad range of severity was allowed to optimize the generalization of the results. Moreover, a brief analysis of the results of less severely/more severely affected participants gave no indication that any differences arose related to the accuracy of the Activ8. In future research, to increase ecological validity, activities should be self-chosen by the patient in a free-living environment and not be imposed by a pre-set activity protocol. 

## 5. Conclusions

The Activ8 Physical Activity Monitor can be used to measure physical behavior during rehabilitation after a stroke. It detects the class “upright position”, “lying/sitting” and “cycling” in a sufficiently accurate way during basic activities and daily-life activities performed in a laboratory or at home. The components of the class “upright position”, that is, “standing” and “walking/running”, were detected with sufficient accuracy during basic activities. The detection of these components during daily-life activities needs further study.

## Figures and Tables

**Table 1:** The pre-set activities of the basic activities protocol and of the daily-life activities protocol.

Basic Activities Protocol	Daily-Life Activities Protocol
Lying (9, 7)	Sitting with upper limb activity ^◊^ (18, 10)
Sitting * (17, 10)	Standing with upper limb activity ^◊^ (13, 10)
Standing * (14, 9)	Walking while carrying an object (11, 2, 2)
Walking overground ^‡^ (16, 6, 3)	Hanging laundry (13, 1)
Walking on treadmill ^‡^ (3, 0)	Packing a bag (12, 5)
Staircase walking (6, 7)	Kitchen activities (12, 9)
Cycling ^‡^ (13, 3, 2)	Personal care activities (13, 1)
	Throwing a ball (8, 0)
	Vacuuming (12, 5)

Numbers between brackets are the number of participants that performed the activity (in the laboratory, at home inside, at home outside). * indicates activities performed twice; ^‡^ indicates activities performed at a comfortable pace and, if possible, also at slower and faster speeds; ^◊^ indicates the sitting or standing body posture while performing multiple functional activities (for example, writing, eating, getting dressed).

**Table 2:** The video data, Activ8 output, time difference (absolute in seconds and as a percentage), and agreement (overall and per protocol).

		Video (s)	Activ8 (s)	Time Difference (s) *	Time Difference (%)	Agreement (%)
Overall	Upright	20,239	19,464	−775	−3.8	92.6
	Lying/sitting	12,754	13,323	569	4.5	95.3
	Cycling	3163	3369	206	6.5	99.4
Basic activities in laboratory	Upright	5651	5624	−27	−0.5	97.6
	*Standing*	*1827*	*2088*	*261*	*14.3*	*95.9*
	*Walking/running*	*3824*	*3536*	*−288*	*−7.5*	*91.5*
	Lying/sitting	3407	3411	4	0.1	97.9
	Cycling	2426	2449	23	1.0	98.6
Basic activities at home	Upright	2967	2878	−89	−3.0	92.9
	*Standing*	*1257*	*1273*	*16*	*1.3*	*89.7*
	*Walking/running*	*1710*	*1605*	*−105*	*−6.1*	*90.4*
	Lying/sitting	2631	2588	−43	−1.6	96.4
	Cycling	737	869	132	17.9	100
Daily activities in Laboratory ^‡^	Upright	6984	6648	−336	−4.8	95.2
	Lying/sitting	3548	3856	308	8.7	87.9
	Cycling	0	28	28	N/A	N/A
Daily activities at Home ^‡^	Upright	4637	4314	−323	−7.0	82.2
	Lying/sitting	3168	3468	300	9.5	100
	Cycling	0	23	23	N/A	N/A

* Calculated as Activ8 minus video recording. Negative value: Activ8 underestimates, positive value: Activ8 overestimates. ^‡^ In daily life activities, the “upright position” was not subdivided into the classes “standing” and “walking/running”, because of the aforementioned issue of not being able to validly allocate the video class “standing with leg movements” to the “standing” or “walking” video class during daily-life activities.

**Table 3:** The registered video and the Activ8 time of daily-life activities (Part A) and free-choice activities (Part B).

Activity	Total (s)	Postures/Movements	Video (s)	Activ8 (s)
Part A				
Hanging laundry	1078	Sitting	0	53
		Standing	865	873
		Standing while moving legs	209	0
		Walking	4	152
Kitchen activities	1707	Sitting	259	256
		Standing	1065	954
		Standing while moving legs	332	0
		Walking	51	494
		Cycling	0	3
Throwing a ball	593	Sitting	75	75
		Standing	455	414
		Standing while moving legs	51	0
		Walking	12	104
Vacuuming	829	Sitting	0	29
		Standing	369	375
		Standing while moving legs	456	0
		Walking	4	425
Part B				
Wheelchair driving with leg propulsion	646	Sitting	646	196
		Cycling	0	450
Using a stair lift	192	Sitting	192	192
Fitness activities	23	Standing	16	2
		Standing while moving legs	4	0
		Walking	3	21
